# Correlation between band gap, dielectric constant, Young’s modulus and melting temperature of GaN nanocrystals and their size and shape dependences

**DOI:** 10.1038/srep16939

**Published:** 2015-11-19

**Authors:** Haiming Lu, Xiangkang Meng

**Affiliations:** 1 National Laboratory of Solid State Microstructures, Collaborative Innovation Center of Advanced Microstructures, College of Engineering and Applied Sciences, Institute of Materials Engineering, Nanjing University, Jiangsu 210093, People’s Republic of China

## Abstract

With structural miniaturization down to the nanoscale, the detectable parameters of materials no longer remain constant but become tunable. For GaN nanocrystals example, the band gap increases while the dielectric constant, Young’s modulus and melting temperature decrease with decreasing the solid size. Herein, we developed the models to describe the size and shape dependences of these seemingly uncorrelated parameters for GaN nanocrystals, based on our established thermodynamic model for cohesive energy of metallic nanocrystals. Consistency between our theoretical predictions and the corresponding experimental or simulated results confirms the accuracy of the developed models and indicates the essentiality of cohesive energy in describing the effects of size and shape on the physicochemical properties of different low-dimensional systems.

GaN and their alloys have been become prominent wide band gap semiconductors due to their applications in optoelectronics and high-power electronics^1^,^2^. Bulk GaN exhibits continuous light emission from near-infrared to ultraviolet region, excellent thermal conductivity, chemical inertness and hardness, and high thermal stability. With the development of nanotechnology, low-dimensional forms of GaN and their alloys are intriguing structures for nanophotonics^3^,^4^,^5^. The possibility of improving light output power, quantum confinement, strain releasing effect, correlated photon emission and photonic crystal effects makes one-dimensional GaN nanomaterials potential contenders for nanoLEDs, nanogenerators and nanotransistors, which has been proved to be useful for information storage, optical interconnects, photocatalysis and general illumination^6^,^7^,^8^,^9^,^10^,^11^,^12^,^13^,^14^.

For GaN, the parameters including the melting temperature *T*_m_, band gap *E*_g_, Young’s modulus *Y* and dielectric constant *ε* are fundamentally important to devise devices. Conventionally, these detectable parameters are treated as constant for a bulk specimen and they are less correlated. However, at the nanoscale, these parameters become tunable due to the size and shape dependences, and the change trends with size and shape are different from one another. Numerous computer simulations and experimental studies have found that the *E*_g_ increase while the *T*_m_, *Y* and *ε* decrease with the decreasing size^15^,^16^,^17^,^18^,^19^,^20^,^21^,^22^,^23^. In order to understand these unusual properties of nanomaterials, a lot of theoretical models have also been proposed from various perspectives. For the size-induced *T*_m_ depression, the liquid drop model, latent heat model and bond energy model have been proposed^24^,^25^,^26^. The size-induced *E*_g_ expansion can be explained in terms of the quantum confinement premise^27^. The size-induced *Y* variation is generally interpreted using the models of surface tension, surface relaxation and surface reconstruction^28^,^29^,^30^. The *ε* suppression is thought to result from the breaking of surface polarizable bonds^31^. Guisbiers *et al*. have expressed the *T*_m_ and *E*_g_ of nanomaterials as functions of its diameter and the shape parameter α_shape_ = *AD*(γ_s_ − γ_l_)/(*VH*_m,∞_) where *D*, *A*, *V* are the diameter, surface area and volume of the nanomaterials, γ_s_ and γ_l_ are the surface energy in the solid and liquid phases, and *H*_m,∞_ is the bulk melting enthalpy^32^. Zhang also introduced a core-surface (CS) model to explain the size effects on the *Y* and *ε* where the nanowires is modelled as a composite beam consisting of the core section of the bulk material and the surface layer^23^. Generally, the size effect is thought to originate from the surface-to-volume ratio effect and the statistic followed by the particles involved in the material property^33^. However, the above-mentioned different theoretical interpretations imply that there are different rules governing the size dependences of these physical parameters.

Here, we attempted to correlate, formulate and quantify these above-mentioned seemingly uncorrelated parameters of GaN nanocrystal and understand the common origin of their size and shape dependences, based on our developed size-, shape-, and dimensionality-dependent cohesive energy or melting temperature model for elemental nanocrystals, which has been deduced as^34^,


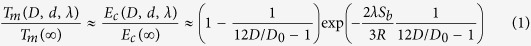


where *E*_c_, *R* and *S*_b_ are the cohesive energy, ideal gas constant and bulk solid-vapor transition entropy. *D*_0_ = 2(3 − *d*)*h* is a critical size where all atoms of crystal are located on its surface with *d* and *h* being the dimensionality and atomic diameter or the nearest spacing^34^. The shape factor *λ* describes the shape effect on the ratio of surface atoms to the total atoms *δ*, which can be determined as the ratio of *δ* between nanocrystals with other shape and those with basal shape (e.g. cylindrical nanowire or spherical nanoparticle)^34^,


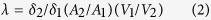


where the subscripts 1 and 2 denote the nanocrystals with basal shape and other shapes. The validity of Eq. (1) has been verified by available experimental, molecular dynamics (MD) simulation and other theoretical results for metallic nanocrystals (e.g. Au, Ag and Ni), molecular nanocrystals (e.g. Ar) and covalent nanocrystals (e.g. Si)^34^. Thus, we assume that Eq. (1) is able to predict the melting behavior of GaN nanocrystals.

Figure. 1 illustrates the dependence of the normalized melting temperature on the thickness *t* of hexagonal GaN nanotubes calculated by Eq. (1) where the inner radius *r*_in_ of nanotube is set as 1.20 nm. As a comparison, available MD simulations results^15^ are also listed. Since a periodic boundary condition has been applied in the axial direction^15^, the influence of two end faces is thus negligible. Moreover, the melting of nanotubes starts from the outer surface and proceeds towards the inner surface^15^, *A*_2_ in Eq. (2) is thus equal to the outer surface area rather than the sum of outer and inner surface area. In this case, *λ* ≈ 2(*r*_in_ + *t*)^2^/[3^1/2^*t*(2*r*_in_ + *t*)] can be obtained in terms of Eq. (2). Note that the melting temperature of bulk GaN is not well known because of experimental difficulties related to the very high melting temperature and the overpressure of N_2_ necessary to prevent decomposition before melting. Experiments in a high-pressure anvil cell indicated that GaN does not melt at a temperature up to 2573 K under 68 kbars of pressure^35^. Vasil’ev and Gachon correlated the melting temperature and enthalpy of formation of III–V compounds and evaluated the melting point of bulk GaN to be 2570 K^36^. There are a few reports on MD simulations of the melting point of bulk GaN. Nord *et al*. simulated the melting temperature to be 3500 ± 500 K^37^. Using a single-phase or a two-phase MD simulation, the melting temperature of bulk GaN is determined to be 4200 and 3000 K, respectively^15^. Since there is no recognized value of the melting temperature for bulk GaN and the MD simulations results^15^ are presented in Fig. 1 for comparison, the average value 3567 K of three reported MD simulation results is taken as a first-order approximation. As shown in Fig. 1, it is evident that the melting temperature of GaN nanotubes increases with the thickness of the nanotubes and agreements between our model predictions and MD results can be found, which confirms our assumption that Eq. (1) can also be used to predict the melting behavior of GaN nanocrystals.

## Formula

To deduce the size dependent band gap of GaN, a well-known Arrhenius expression for the size and temperature dependent electrical conductivity *μ*(*D*,*T*) is introduced,


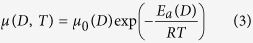


where *μ*_0_ denotes a pre-exponential constant. The activation energy for electrical migration is *E*_a_ = *E*_c_ − *E*_F_ with *E*_c_ and *E*_F_ being the conduction-band energy and the Fermi energy. In many semiconductors, *E*_F_ is near mid-gap and thus *E*_a_ ≈ *E*_g_/2^38^. If the change of *E*_g_ is supposed to be proportional to the change of *E*_a_, there is Δ*E*_g_(*D*)/*E*_g_(∞) = |Δ*E*_a_(*D*)/*E*_a_(∞)| where Δ denotes the change. Assuming that the electrical conductivity at the melting temperature is the same, independent of the melting temperature and therefore independent of the size, one can obtain the expression


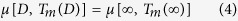


Combining Eq. (3) with Eq. (4), there is


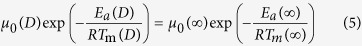


Because the effect of the exponential term of exp[−*E*_a_(*D*)/(*RT*)] on *μ*(*D*,*T*) is much stronger than the effect of *μ*_0_(*D*), as a first-order approximation, one can assume that *μ*_0_(*D*) ≈ *μ*_0_(∞). Therefore, there is


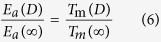


and then


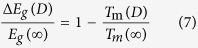


The dielectric constant results from electronic polarization or electron migration from the lower valence band to the upper conduction band. This process is subject to the selection rule of energy and momentum conservation, which determines the optical response of semiconductors and reflects how strongly the valence electrons couple with the excited conduction electrons^39^. Thus, the dielectric constant of a semiconductor is directly related to its band gap at room temperature. Extending the relationship of *ε*(∞) = *χ*(∞) + 1 and the approximation relation of *χ*(∞) ∝ [*E*_g_(∞)]^−2^ into nanoscale with *χ* being the electric susceptibility^39^, we have


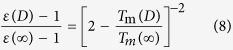


It is known that the Young modulus is fundamentally related to the interatomic bonding and is thus influenced by modifications of the atomic environment. Semi-empirical methods to correlate the Young modulus and the surface thermodynamic properties are always possible. For example, the Young modulus is linked to the surface energy γ_sv_ with the following expression of 
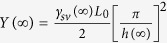
 where *L*_0_ denotes the equilibrium interplanar spacing normal to a surface of the solid^40^. Since the GaN nanocrystal remains the wurtzite structure which is the same as the corresponding bulk, the expression may thus be extended to nanometer size, namely


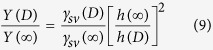


Although the nanocrystal has lattice contraction induced by the large surface-to-volume ratio, *ab initio* density functional investigations of the atomic structure of GaN nanocrystals with diameters ranging from 2.28 to 1.1 nm found that the contraction of Ga-N bond length at the surface is only 0.7–0.9% in comparison with the bulk^20^. Considering that the *Y*(*D*) depression reaches about 40% when the cross-sectional size of square nanowires is reduced to 1 nm^23^, we can concluded that the contribution of *h*(*D*) on *Y*(*D*) is negligible in this case as a first order approximation to simplify the derivations and calculations. On the other hand, the surface energy denotes the bond energy difference between surface atoms and interior ones while the melting temperature is also directly proportional to the bond strength, the size dependence of the surface energy has thus been deduced to be the same as that of the melting temperature^41^. Combining the above discussions, we can have


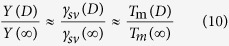


## Results

Figure. 2a compares the Δ*E*_g_(*D*) functions of hexagonal GaN nanowires between the model prediction of Eq. (7) combined with Eq. (1) and the computer simulation results^17^,^19^,^21^ where agreements can be found, noted that the *λ* value for hexagonal GaN nanowires can be determined as 2/3^1/2^ according to Eq. (2). It is known that different simulation methods result in different results, and we choose the DFT calculations employing the screened Heyd-Scuseria-Ernzerhof 06 hybrid functional rather than the local density approximation for comparison since the former gave more accurate prediction of *E*_g_(∞) than the latter^19^. The band gap of GaN nanocrystals is not only size-dependent but also shape-dependent. Figure. 2b shows the Δ*E*_g_(*D*) functions of hexagonal and triangular GaN nanowires in terms of Eq. (7) where the *λ* value for triangular nanowires can be determined as 3^1/2^ according to Eq. (2). These two kinds of GaN nanowires show an increase in the band gap with the decreasing diameter and the band gap of triangular nanowires is larger than that of hexagonal nanowires for a given wire diameter. This trend is understandable since the triangular nanowires are less stable and thus the occupied edge-induced states are at a higher energy compared to the hexagonal nanowires. As a comparison, available DFT calculations employing the DMOL^3^ rather than SIESTA code are listed since the predictions of DMOL^3^ for bulk properties are closer to the experimental results than those of SIESTA^18^. Noted that the calculated band gap of bulk GaN by DMOL^3^ is only 2.58 eV rather than the experimental result of 3.50 eV, *E*_g_(∞) = 2.58 eV is thus taken in our calculations. As shown in Fig. 2b, the model predictions are in good agreements with the corresponding DFT simulation results.

Figure 2c presents the Δ*E*_g_(*D*) functions of spherical GaN nanoparticles and cylindrical nanowires in terms of Eq. (7) with *λ* = 1 according to the definition of the shape factor. As a comparison, available DFT simulations results^20^ are also listed, which agree with the corresponding model predictions. As shown in Fig. 2c, the relative band gap of the nanowires is always smaller than that of the nanoparticles. Considering the mathematical relation of exp(−*x*) ≈ 1 − *x* when *x* is small enough, as a first order approximation, Eq. (7) combined with Eq. (1) can be simplified as


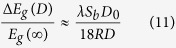


Since *D*_0_ = 2(3 − *d*)*h* with the dimensionality *d* being 0 and 1 for the nanoparticles and nanowires, the ratio of the relative band gap between the nanowires and the nanoparticles is about 2/3, which is similar to the results (0.584 for large size and 2/3 for small size) obtained from an effective mass approximation calculations^42^ and 0.62 obtained from the experimental investigations^43^.

In Fig. 2a–c, the employed simulation results correspond to the nanowires with infinite length^17^,^18^,^19^,^20^,^21^,^22^,^23^. However, not only the diameter but also the length has an influence on how strong the size effect will be, when the length *L* of the nanowires is comparable to the diameter. In this case, *A*_2_ = 2π(*D*/2)^2^ + π*DL* and *V*_2_ = π(*D*/2)^2^*L* for cylindrical nanowires with finite length *L*. While for cylindrical nanowires with infinite length *L*′, the surface areas of the top and bottom of nanowire are negligible as a first order approximation due to the length *L*′ » *D*, namely *A*_1_ ≈ π*DL*′ and *V*_1_ = π(*D*/2)^2^*L*′. In terms of Eq. (2), the shape factor *λ* can be determined as *λ* ≈ 1 + *D*/(2*L*). As shown in Fig. 2c, the Δ*E*_g_(*D*) functions of cylindrical GaN nanowires with *L*/*D* = 3 is presented (the blue line) based on Eq. (7). It can be found that the size effect of nanowires with finite length is stronger than that of nanowires with infinite length while smaller than that of nanoparticles when the diameter is the same.

In addition to the size, the band gap of semiconductors can be tuned by alloying. It is reported that the semiconductor nanoalloys have luminescent properties comparable to or even better than the best-reported binary semiconductor nanocrystals and they are promising materials for optoelectronic devices^5^,^22^. The experimental and theoretical results have found that the composition-dependent band gap *E*_g_(*x*,∞) of the bulk varies monotonically but not linearly with the composition *x* over the whole range of composition^5^,^22^,^32^. On the basis of assumption that a bulk ternary or pseudo-binary semiconductor compound alloy is a regular solution of components, *E*_g_(*x*,∞) of A_*x*_B_1-*x*_C semiconductor is often described by an empirical expression of *E*_g_(*x*,∞) = *xE*_g_(AC,∞) + (1 − *x*)*E*_g_(BC,∞) + *bx*(1 − *x*) where AC and BC are two binary compounds and *b* is the bowing parameter of A_*x*_B_1-*x*_C^22^,^32^. The value of *b* is a measure of the fluctuation magnitude of the crystal field or the nonlinear effect caused by the anisotropic nature of binding, which is difficult to measure experimentally. Guisbiers *et al*. have predicted the Al_*x*_Ga_1-*x*_N energy band gap with composition for the bulk and nanoparticles with *D* = 4 nm through firstly calculating the theoretical phase diagram. Alternatively, the Fox equation can be used to calculate the composition-dependent band gap when the structure and optoelectronic properties of the binary semiconductors are similar^44^. Extending the Fox equation into nanoscale, we have


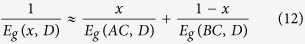


where *E*_g_(AC,*D*) and *E*_g_(BC,*D*) can be determined by Eq. (7).

Figure. 2d plots the *E*_g_(*x*) as a function of constituent stoichiometry *x* for Al_*x*_Ga_1-*x*_N nanowires with *D* = 15 nm in terms of Eqs. (12) and (7), where the experimental data are also included for comparison^22^. It can be observed that the *E*_g_(*x*) plot has downward shift and *E*_g_(*x*) increases with the increasing *x* and the prediction are consistent with the experimental results. The agreement confirms the advantage of Eq. (12) in comparison with the empirical expression that there is no adjustable parameter in the equation and hence it substantially simplifies the calculation of *E*_g_(*x*,*D*) for semiconductor nanoalloys. As shown in Fig. 2a–d, both size and composition can increase the band gap of semiconductor. Thus, in order to raise the band gap of narrow-gapped semiconductor, applying alloying nanocrystals is a better way compared to semiconductor elements or compounds just by decreasing size.

Based on Eqs. (8) and (10), we calculate the reduced *ε*(*D*) and *Y*(*D*) functions of square GaN nanowires as shown in Fig. 3, where *λ* = 1 is determined for square GaN nanowires according to Eq. (2). Clearly, the calculated *ε*(*D*) and *Y*(*D*) values correspond to the MD simulations results^23^. In detail, *ε*(*D*) and *Y*(*D*) decreases with the decreasing cross-sectional size and the drop becomes significant when *D* < 2 nm. Similar to the blue line presented in Fig. 2c, the length effect on the dielectric constant and Young’s modulus should also be include when the length *L* of the nanowires is comparable to the diameter. In this case, *A*_2_ = 2*D*^2^+4*DL* and *V*_2_ = *D*^2^*L* for square nanowires with finite length *L* and then the shape factor *λ* can be determined as *λ* ≈ 1 + *D*/(2*L*) in terms of Eq. (2). As shown in Fig. 3, the *ε*(*D*) and *Y*(*D*) functions of square GaN nanowires with *L*/*D* = 3 is presented (the blue line) based on Eqs. (8) and (10). It can be found that the *ε*(*D*) and *Y*(*D*) suppression of nanowires with finite length is stronger than that of nanowires with infinite length and the size effect increases with the decrease of the *L*/*D* value.

**Figure 1:**
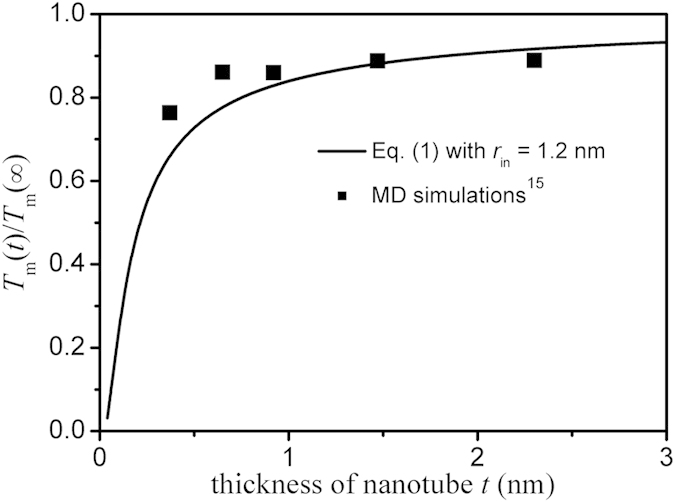
Comparison of *T*_m_*(t)/T*_m_(∞) function of GaN nanotubes with (100) facets between model prediction based on Eq. (1) with D_0_ = 4*h* and MD simulation results^15^, where the inner radius *r*_in_ of nanotube is set as 1.20 nm. *h* = *a*^2^/(6*c*) + *c*/4 ≈ 0.162 nm for wurtzite structure with *a* = 0.319 nm and *c* = 0.519 nm being the lattice constants^18^. Since the bulk solid-vapor transition entropy for GaN is unavailable, *S*_b_ ≈ 13*R* is taken as a first-order approximation, which equals to that of the mean value of most elements (70–150 J/mol-K)^53^.

It should be mentioned that the simulations results shown in Figs 1 and 3 were obtained from the classical MD simulation based on Newton mechanics. The agreements among available experimental data, classical MD simulation results and theoretical predictions of Eq. (1) shown in ref. 34 indicate the reliability of the classical MD simulation when the size is reduced to several nanometers. Moreover, even though there are some errors in simulating the physical parameters, it does not affect the numerical result in the present approach since we are considering the relative changes.

## Discussions

**Figure 2:**
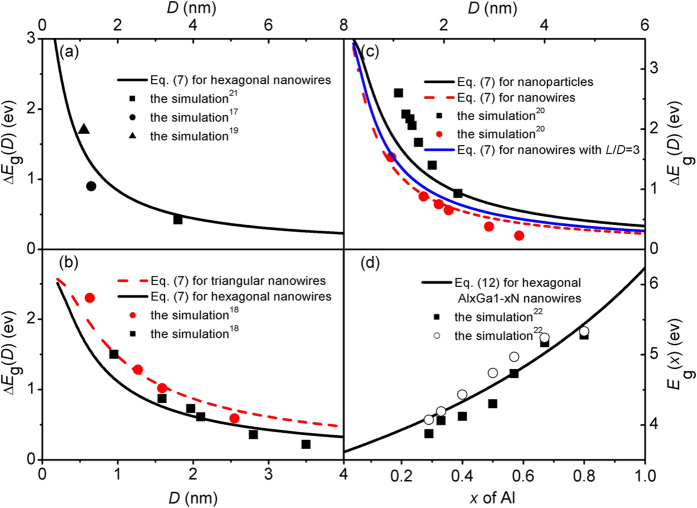
Δ*E*_g_(*D*) functions of GaN nanoparticles and nanowires and *E*_g_(*x*) functions of Al_*x*_Ga_1-*x*_N nanowires with *D* = 15 nm where the curves denote the model predictions of Eqs. (7) and (12) while the symbols are the computer simulation results and the experimental data^17^,^18^,^19^,^20^,^21^,^22^. For AlN, *E*_g_(∞) = 6.03 eV and *h* = *a*^2^/(6*c*)+*c*/4 ≈ 0.159 nm with *a* = 0.311 nm and *c* = 0.508 nm^54^.

**Figure 3:**
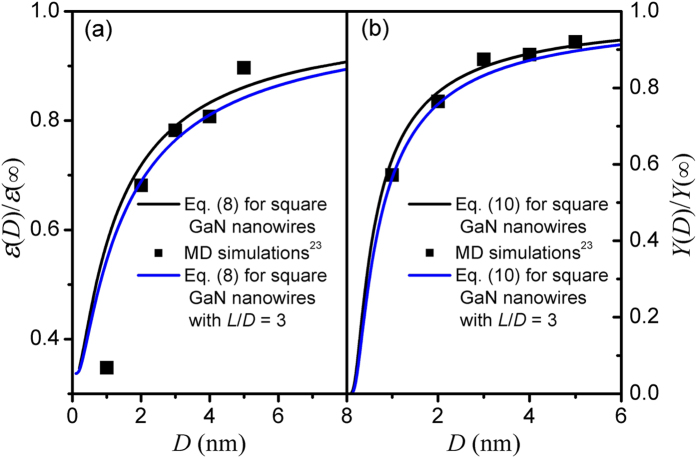
(**a**) *ε*(*D*)/*ε*(∞) function and (**b**) *Y*(*D*)/*Y*(∞) function of square GaN nanowires. The lines are plotted in terms of Eq. (8) with *ε*(∞) = 8.63 and Eq. (10) with *Y*(∞) = 327 GPa where the symbols are the corresponding MD simulations results^23^.

The agreements shown in Figs 1, 2, 3 not only confirm the accuracy and validity of our developed models but also indicate that there must be the common origin of the size dependences of these seemingly uncorrelated parameters. Pauling and Goldschmidt indicated that, if the coordination number of an atom were reduced, the ionic or metallic diameter of the atom would shrink spontaneously^45^. A bond-order-length-strength correlation mechanism also indicated that imperfections in atom coordination would cause the remaining bonds of under-coordinated atom to contract in association with a gain in strength of single bonds^45^, noted that the coordination imperfection can originate from the changes of the size and shape. However, because of the bond-order loss, the cohesive energy (which is the sum of single-bond energies over all of the coordinates of a specific atom) of an under-coordinated atom will drop^45^. As the result, the cohesive energy suppression induced local quantum entrapment perturbs the Hamiltonian that determines the band gap (band-gap expansion) and hence the process of electron polarization consequently (dielectric constant suppression) since the dielectric suppression is thought to result from the breaking of surface polarizable bonds^46^. Zhou and Huang have proposed that whether the Young’s modulus is softer or stiffer depends on the competition of bond loss and bond saturation^47^, where the bond loss originates from the cohesive energy suppression while the bond saturation is induced by the electron redistribution. The agreement between the model prediction and the corresponding MD simulation results shown in Fig. 3b indicates that the bond loss is too much to be compensated by the bond saturation, leading to the reduction of Young’s modulus of GaN nanowires. Based on Lindemann’s criterion of melting, the melting is related to the lattice vibration where the latter is determined by the cohesive energy^34^. Moreover, Li *et al*. ascribed the melting to diffusion of the local clusters, which would lead to some new defects in nanocrystals, noting that these defects would cause the decrease of the cohesive energy and thus accelerate the melting^48^. Together with the above discussions and the fact that Eq. (1) is established based on the size-dependent cohesive energy model, we can conclude it is the coordination-imperfection induced cohesive energy suppression responsible for the size and shape trends of the melting temperature, band gap, dielectric constant and Young’s modulus.

The agreement between our developed models and the observations not only confirms the validity of our models but also provides guidelines for III–V semiconductor nanomaterials and device design. Together with previous works on size dependences of thermal stability and surface energy of nanoparticles and nanocavities^24^,^41^,^49^,^50^, surface tension of liquid droplets^49^, thermal conductivity and diffusivity of semiconductor nanocrystals^51^, and catalytic activation energy of metallic nanocrystals^52^, the work here indicates the essentiality of cohesive energy in describing the effects of size and shape on the physicochemical properties of different low-dimensional systems.

## Additional Information

**How to cite this article**: Lu, H. M. and Meng, X. K. Correlation between band gap, dielectric constant, Young’s modulus and melting temperature of GaN nanocrystals and their size and shape dependences. *Sci. Rep*. **5**, 16939; doi: 10.1038/srep16939 (2015).
